# 44499 Heterogeneity of treatment effect among patients with type 2 diabetes and body mass index >=27kg/m^2 in the Jump Start Study

**DOI:** 10.1017/cts.2021.525

**Published:** 2021-03-30

**Authors:** Elizabeth A. Kobe, Mathew J. Crowley, Amy Jeffreys, William S. Yancy, Jennifer Zervakis, David E. Edelman, Cynthia J. Coffman

**Affiliations:** 1 Duke University School of Medicine; 2 Durham Veterans Affairs Center of Innovation to Accelerate Discovery and Practice Transformation (ADAPT), Duke University School of Medicine; 3 Durham Veterans Affairs Center of Innovation to Accelerate Discovery and Practice Transformation (ADAPT)

## Abstract

ABSTRACT IMPACT: This is the first study to use QUINT analyses to examine heterogeneity of treatment effect for group medical visits among individuals with type 2 diabetes. QUINT is a data driven method that assumes no a priori assumptions regarding effect moderators - an important step in the path towards personalized medicine. OBJECTIVES/GOALS: To examine heterogeneity of treatment effect (HTE) in Jump Start, a trial that compared the effectiveness of group medical visits (GMVs) focused on medication management only versus the addition of intensive weight management (WM) on glycemic control for patients with type 2 diabetes and body mass index >=27kg/m^2. METHODS/STUDY POPULATION: Jump Start patients (n=263) were randomized to a GMV-based medication management plus low carbohydrate diet-focused WM program (WM/GMV; n = 127) or GMV-based medication management only (GMV; n = 136) for diabetes control. We used QUalitative INteraction Trees (QUINT), a tree-based clustering method, to determine if there were subgroups of patients who derived greater benefit from either WM/GMV or GMV. Subgroup predictors included 32 baseline demographic, clinical, and psychosocial factors. Outcome was hemoglobin A1c (HbA1c). We conducted internal validation via bootstrap resampling to estimate bias in the range of mean outcome differences among arms. RESULTS/ANTICIPATED RESULTS: QUINT analyses indicated that for patients who had not previously attempted weight loss, WM/GMV resulted in better glycemic control than GMV alone (mean difference in HbA1c improvement = 1.48%). For patients who had previously attempted weight loss and had lower cholesterol and blood urea nitrogen levels, GMV alone was better than WM/GMV (mean difference in HbA1c improvement = 1.51%). Internal validation resulted in moderate corrections in the mean HbA1c differences between arms; however, differences remained in the clinically significant range. DISCUSSION/SIGNIFICANCE OF FINDINGS: Among patients with diabetes and BMI>=27kg/m^2, a low-carbohydrate, weight loss focus may better improve HbA1c in those who have never attempted weight loss. A medication management focus may be better in those who have attempted weight loss and have lower cholesterol and blood urea nitrogen.

